# *N*′-[(*E*)-4-Bromo­benzyl­idene]-1-benzofuran-2-carbohydrazide monohydrate

**DOI:** 10.1107/S1600536812026724

**Published:** 2012-06-16

**Authors:** Hoong-Kun Fun, Ching Kheng Quah, Nitinchandra, Balakrishna Kalluraya, M. Babu

**Affiliations:** a X-ray Crystallography Unit, School of Physics, Universiti Sains Malaysia, 11800 USM, Penang, Malaysia; b Department of Studies in Chemistry, Mangalore University, Mangalagangotri, Mangalore 574 199, India

## Abstract

The title compound, C_16_H_11_BrN_2_O_2_·H_2_O, exists in a *trans* conformation with respect to the N=C bond [1.2815 (14) Å] and the benzofuran ring system forms a dihedral angle of 2.96 (5)° with the benzene ring. In the crystal, the ketone O atom accepts two O—H⋯O and one C—H⋯O hydrogen bond, and the water O atom accepts an N—H⋯O inter­action. Together, these lead to infinite layers lying parallel to (100).

## Related literature

For related structures and background to the biological activity of hydrazones, see: Fun *et al.* (2012*a* ▶,*b* ▶). For the stability of the temperature controller used in the data collection, see Cosier & Glazer (1986 ▶).
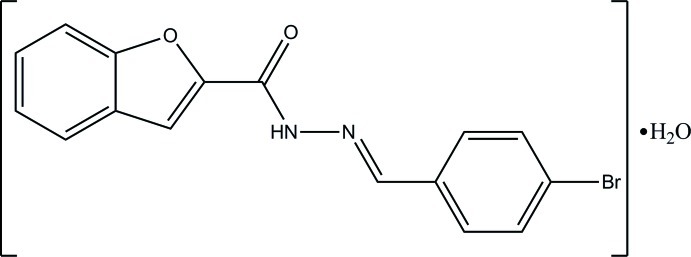


## Experimental

### 

#### Crystal data

C_16_H_11_BrN_2_O_2_·H_2_O
*M* *_r_* = 361.19
Monoclinic, 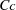

*a* = 25.0594 (4) Å
*b* = 4.6718 (1) Å
*c* = 12.6166 (2) Å
β = 99.175 (1)°
*V* = 1458.16 (5) Å^3^
*Z* = 4
Mo *K*α radiation
μ = 2.83 mm^−1^
*T* = 100 K
0.41 × 0.22 × 0.13 mm

#### Data collection

Bruker SMART APEXII CCD diffractometer
Absorption correction: multi-scan (*SADABS*; Bruker, 2009 ▶) *T*_min_ = 0.390, *T*_max_ = 0.708
22903 measured reflections
6458 independent reflections
6000 reflections with *I* > 2σ(*I*)
*R*_int_ = 0.024

#### Refinement

*R*[*F* ^2^ > 2σ(*F* ^2^)] = 0.021
*wR*(*F* ^2^) = 0.048
*S* = 1.00
6458 reflections
205 parameters
2 restraints
H atoms treated by a mixture of independent and constrained refinement
Δρ_max_ = 0.55 e Å^−3^
Δρ_min_ = −0.24 e Å^−3^
Absolute structure: Flack (1983 ▶), 3029 Friedel pairs
Flack parameter: 0.002 (3)

### 

Data collection: *APEX2* (Bruker, 2009 ▶); cell refinement: *SAINT* (Bruker, 2009 ▶); data reduction: *SAINT*; program(s) used to solve structure: *SHELXTL* (Sheldrick, 2008 ▶); program(s) used to refine structure: *SHELXTL*; molecular graphics: *SHELXTL*; software used to prepare material for publication: *SHELXTL* and *PLATON* (Spek, 2009 ▶).

## Supplementary Material

Crystal structure: contains datablock(s) global, I. DOI: 10.1107/S1600536812026724/hb6850sup1.cif

Structure factors: contains datablock(s) I. DOI: 10.1107/S1600536812026724/hb6850Isup2.hkl

Supplementary material file. DOI: 10.1107/S1600536812026724/hb6850Isup3.cml

Additional supplementary materials:  crystallographic information; 3D view; checkCIF report

## Figures and Tables

**Table 1:** Hydrogen-bond geometry (Å, °)

*D*—H⋯*A*	*D*—H	H⋯*A*	*D*⋯*A*	*D*—H⋯*A*
O1*W*—H2*W*1⋯O2^i^	0.85	2.00	2.7932 (11)	157
O1*W*—H1*W*1⋯O2^ii^	0.82	2.10	2.8987 (11)	167
N1—H1*N*1⋯O1*W* ^iii^	0.890 (17)	1.947 (18)	2.8108 (13)	163.4 (17)
C6—H6*A*⋯O2^iv^	0.93	2.53	3.3418 (18)	146

Symmetry codes: (i) 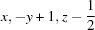; (ii) 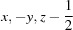; (iii) 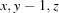; (iv) 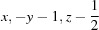.

**Fig. 1.**
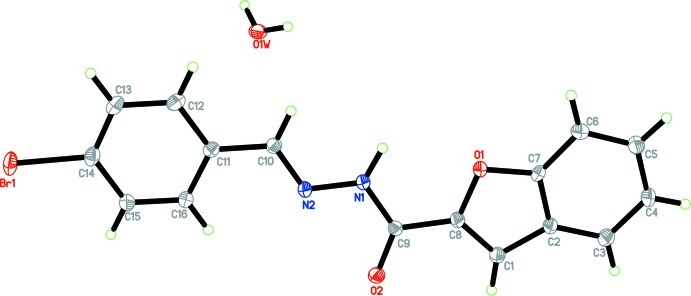
The molecular structure of the title compound showing 50% probability displacement ellipsoids for non-H atoms.

**Fig. 2.**
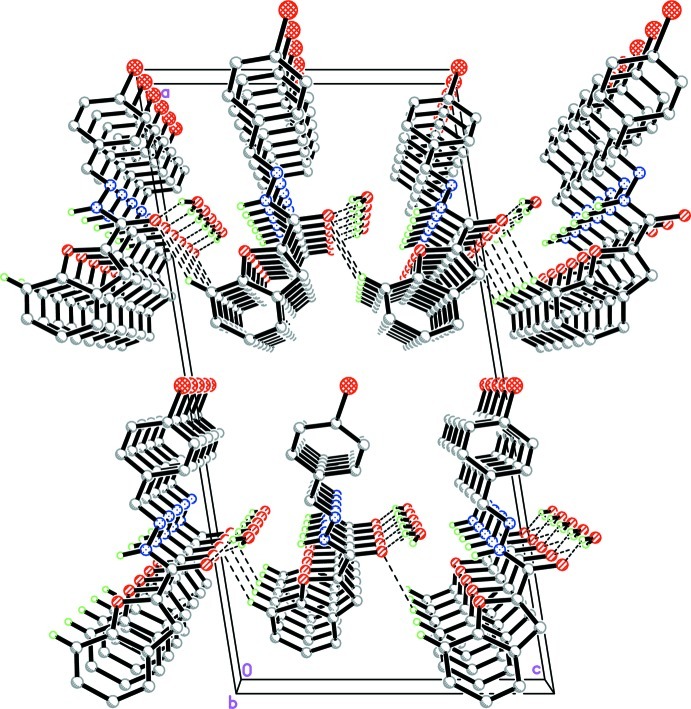
The crystal structure of the title compound, viewed along the b axis. H atoms not involved in hydrogen bonds (dashed lines) have been omitted for clarity.

## supplementary crystallographic information

### Comment

As part of our ongoing synthetic and structural studies of hydrazones with possible biological activities (Fun *et al.*, 2012*a*,*b*), the title compound, (I), was synthesized and its crystal structure is now reported.

The title compound (Fig. 1) crystallises as a hydrate and exists in a *trans* conformation with respect to the N2═C10 bond [1.2815 (14) Å]. The benzofuran ring system (O1/C1-C8, r.m.s deviation = 0.016 Å) forms a dihedral angle of 2.96 (5)° with the benzene ring (C11-C16). Bond lengths and angles are comparable to those in related structures (Fun *et al.*, 2012*a*, 2012*b*)

In the crystal (Fig.2), molecules are linked *via* O1W–H2W1···O2, O1W–H1W1···O2 and C6–H6A···O2 trifurcated acceptor bonds (Table 1) and together with N1–H1N1···O1W hydrogen bonds form two-dimensional arrays parallel to (100).

### Experimental

The title compound was obtained by refluxing a mixture of 1-benzofuran-2-carbohydrazide (0.01 mol), 4-bromobenzaldehyde (0.01 mol) in ethanol (30 ml) and 3 drops of concentrated sulfuric acid for 1 h. Excess ethanol was removed from the reaction mixture under reduced pressure. The solid product obtained was filtered, washed with ethanol and dried. Colourless blocks were obtained by slow evaporation of an ethanol-*N*,*N*-dimethylformamide (DMF) (3:1) solution.

### Refinement

Atom H1N1 was located in a difference Fourier map and refined freely [N1–H1N1 = 0.890 (17) Å]. O-bound H atoms were located in a difference Fourier map and refined using a riding model with O–H = 0.8182 or 0.8477 Å. The rest of hydrogen atoms were positioned geometrically and refined using a riding model with C–H = 0.93 Å and *U*_iso_(H) = 1.2 *U*_eq_(C).

### Crystal data

**Table:**

C_16_H_11_BrN_2_O_2_·H_2_O	*F*(000) = 728
*M**_r_* = 361.19	*D*_x_ = 1.645 Mg m^−^^3^
Monoclinic, *C**c*	Mo *K*α radiation, λ = 0.71073 Å
Hall symbol: C -2yc	Cell parameters from 9978 reflections
*a* = 25.0594 (4) Å	θ = 3.3–35.9°
*b* = 4.6718 (1) Å	µ = 2.83 mm^−^^1^
*c* = 12.6166 (2) Å	*T* = 100 K
β = 99.175 (1)°	Block, colourless
*V* = 1458.16 (5) Å^3^	0.41 × 0.22 × 0.13 mm
*Z* = 4	

### Data collection

**Table:**

Bruker SMART APEXII CCD diffractometer	6458 independent reflections
Radiation source: fine-focus sealed tube	6000 reflections with *I* > 2σ(*I*)
Graphite monochromator	*R*_int_ = 0.024
φ and ω scans	θ_max_ = 35.9°, θ_min_ = 3.3°
Absorption correction: multi-scan (*SADABS*; Bruker, 2009)	*h* = −38→40
*T*_min_ = 0.390, *T*_max_ = 0.708	*k* = −7→7
22903 measured reflections	*l* = −20→20

### Refinement

**Table:**

Refinement on *F*^2^	Secondary atom site location: difference Fourier map
Least-squares matrix: full	Hydrogen site location: inferred from neighbouring sites
*R*[*F*^2^ > 2σ(*F*^2^)] = 0.021	H atoms treated by a mixture of independent and constrained refinement
*wR*(*F*^2^) = 0.048	*w* = 1/[σ^2^(*F*_o_^2^) + (0.0159*P*)^2^] where *P* = (*F*_o_^2^ + 2*F*_c_^2^)/3
*S* = 1.00	(Δ/σ)_max_ = 0.001
6458 reflections	Δρ_max_ = 0.55 e Å^−^^3^
205 parameters	Δρ_min_ = −0.24 e Å^−^^3^
2 restraints	Absolute structure: Flack (1983), 3029 Friedel pairs
Primary atom site location: structure-invariant direct methods	Flack parameter: 0.002 (3)

### Special details

**Table:**

Experimental. The crystal was placed in the cold stream of an Oxford Cryosystems Cobra open-flow nitrogen cryostat (Cosier & Glazer, 1986) operating at 100.0 (1) K.
Geometry. All esds (except the esd in the dihedral angle between two l.s. planes) are estimated using the full covariance matrix. The cell esds are taken into account individually in the estimation of esds in distances, angles and torsion angles; correlations between esds in cell parameters are only used when they are defined by crystal symmetry. An approximate (isotropic) treatment of cell esds is used for estimating esds involving l.s. planes.
Refinement. Refinement of F^2^ against ALL reflections. The weighted R-factor wR and goodness of fit S are based on F^2^, conventional R-factors R are based on F, with F set to zero for negative F^2^. The threshold expression of F^2^ > 2sigma(F^2^) is used only for calculating R-factors(gt) etc. and is not relevant to the choice of reflections for refinement. R-factors based on F^2^ are statistically about twice as large as those based on F, and R- factors based on ALL data will be even larger.

### Fractional atomic coordinates and isotropic or equivalent isotropic displacement parameters (Å^2^)

**Table:**

	*x*	*y*	*z*	*U*_iso_*/*U*_eq_	
Br1	0.493770 (6)	1.100705 (19)	0.508751 (8)	0.02217 (3)	
O1	0.18964 (3)	−0.55368 (16)	0.27425 (6)	0.01388 (14)	
O2	0.23751 (3)	−0.22894 (17)	0.52806 (6)	0.01613 (14)	
N1	0.25775 (4)	−0.12217 (17)	0.36132 (8)	0.01253 (15)	
N2	0.29362 (4)	0.08563 (17)	0.40538 (8)	0.01311 (15)	
C1	0.15501 (5)	−0.6274 (2)	0.42754 (10)	0.0167 (2)	
H1A	0.1499	−0.6159	0.4988	0.020*	
C2	0.12501 (5)	−0.7988 (2)	0.34457 (9)	0.01525 (18)	
C3	0.08115 (5)	−0.9889 (3)	0.33817 (10)	0.0212 (2)	
H3A	0.0646	−1.0253	0.3978	0.025*	
C4	0.06318 (5)	−1.1207 (2)	0.24052 (10)	0.0196 (2)	
H4A	0.0339	−1.2455	0.2343	0.024*	
C5	0.08847 (5)	−1.0683 (2)	0.15094 (11)	0.0174 (2)	
H5A	0.0757	−1.1616	0.0868	0.021*	
C6	0.13193 (7)	−0.8818 (2)	0.15468 (11)	0.0167 (2)	
H6A	0.1489	−0.8486	0.0955	0.020*	
C7	0.14821 (4)	−0.7488 (2)	0.25269 (9)	0.01312 (17)	
C8	0.19229 (4)	−0.4854 (2)	0.38103 (9)	0.01323 (17)	
C9	0.23162 (4)	−0.2696 (2)	0.42951 (9)	0.01253 (17)	
C10	0.32052 (4)	0.2111 (2)	0.34060 (9)	0.01383 (17)	
H10A	0.3147	0.1628	0.2682	0.017*	
C11	0.36048 (6)	0.4314 (2)	0.38081 (11)	0.0136 (2)	
C12	0.39181 (5)	0.5549 (2)	0.31113 (11)	0.0176 (2)	
H12A	0.3860	0.5028	0.2391	0.021*	
C13	0.43169 (5)	0.7550 (2)	0.34769 (10)	0.0184 (2)	
H13A	0.4531	0.8332	0.3012	0.022*	
C14	0.43889 (5)	0.8351 (2)	0.45464 (10)	0.0167 (2)	
C15	0.40692 (5)	0.7233 (2)	0.52486 (9)	0.0175 (2)	
H15A	0.4116	0.7844	0.5959	0.021*	
C16	0.36790 (5)	0.5192 (2)	0.48830 (9)	0.01555 (19)	
H16A	0.3467	0.4410	0.5352	0.019*	
O1W	0.27065 (4)	0.74269 (17)	0.14979 (7)	0.01805 (15)	
H2W1	0.2695	0.8848	0.1077	0.010 (4)*	
H1W1	0.2584	0.5915	0.1236	0.039 (6)*	
H1N1	0.2547 (7)	−0.166 (4)	0.2920 (14)	0.020 (4)*	

### Atomic displacement parameters (Å^2^)

**Table:**

	*U*^11^	*U*^22^	*U*^33^	*U*^12^	*U*^13^	*U*^23^
Br1	0.01396 (4)	0.01463 (4)	0.03562 (7)	−0.00355 (5)	−0.00306 (4)	0.00589 (5)
O1	0.0141 (4)	0.0144 (3)	0.0133 (4)	−0.0033 (2)	0.0027 (3)	−0.0009 (2)
O2	0.0209 (4)	0.0149 (3)	0.0130 (3)	−0.0020 (3)	0.0039 (3)	−0.0014 (2)
N1	0.0136 (4)	0.0121 (3)	0.0119 (4)	−0.0020 (3)	0.0022 (3)	−0.0012 (3)
N2	0.0126 (4)	0.0113 (3)	0.0150 (4)	−0.0019 (3)	0.0009 (3)	−0.0012 (3)
C1	0.0201 (5)	0.0172 (5)	0.0136 (5)	−0.0042 (3)	0.0053 (4)	−0.0019 (3)
C2	0.0153 (5)	0.0148 (4)	0.0160 (5)	−0.0031 (3)	0.0035 (4)	−0.0005 (3)
C3	0.0224 (6)	0.0227 (5)	0.0197 (6)	−0.0093 (4)	0.0069 (4)	−0.0013 (4)
C4	0.0179 (5)	0.0197 (5)	0.0209 (6)	−0.0075 (4)	0.0017 (4)	−0.0007 (4)
C5	0.0183 (5)	0.0177 (4)	0.0152 (5)	−0.0016 (3)	0.0001 (4)	−0.0015 (3)
C6	0.0183 (6)	0.0197 (5)	0.0123 (5)	−0.0014 (4)	0.0028 (4)	0.0003 (4)
C7	0.0119 (4)	0.0116 (4)	0.0159 (5)	−0.0013 (3)	0.0025 (4)	0.0008 (3)
C8	0.0145 (4)	0.0122 (4)	0.0128 (5)	−0.0014 (3)	0.0016 (4)	−0.0019 (3)
C9	0.0127 (4)	0.0111 (4)	0.0139 (5)	0.0001 (3)	0.0022 (4)	−0.0003 (3)
C10	0.0147 (5)	0.0132 (4)	0.0136 (5)	−0.0003 (3)	0.0023 (4)	−0.0001 (3)
C11	0.0130 (5)	0.0121 (4)	0.0157 (5)	0.0017 (3)	0.0023 (4)	0.0023 (3)
C12	0.0190 (5)	0.0172 (4)	0.0176 (5)	−0.0006 (4)	0.0061 (4)	0.0020 (4)
C13	0.0163 (5)	0.0167 (4)	0.0232 (6)	−0.0012 (3)	0.0060 (4)	0.0046 (4)
C14	0.0131 (5)	0.0121 (4)	0.0239 (6)	−0.0006 (3)	0.0004 (4)	0.0041 (3)
C15	0.0170 (5)	0.0168 (4)	0.0178 (5)	−0.0034 (3)	0.0002 (4)	0.0016 (4)
C16	0.0152 (5)	0.0156 (4)	0.0159 (5)	−0.0030 (3)	0.0024 (4)	0.0006 (3)
O1W	0.0251 (4)	0.0160 (3)	0.0129 (4)	−0.0005 (3)	0.0026 (3)	0.0005 (3)

### Geometric parameters (Å, º)

**Table:**

Br1—C14	1.8967 (11)	C5—H5A	0.9300
O1—C7	1.3757 (13)	C6—C7	1.3860 (17)
O1—C8	1.3757 (13)	C6—H6A	0.9300
O2—C9	1.2431 (13)	C8—C9	1.4730 (15)
N1—C9	1.3501 (14)	C10—C11	1.4686 (17)
N1—N2	1.3783 (12)	C10—H10A	0.9300
N1—H1N1	0.890 (17)	C11—C12	1.3938 (18)
N2—C10	1.2815 (14)	C11—C16	1.4006 (18)
C1—C8	1.3537 (16)	C12—C13	1.3923 (17)
C1—C2	1.4323 (16)	C12—H12A	0.9300
C1—H1A	0.9300	C13—C14	1.3841 (18)
C2—C7	1.3970 (15)	C13—H13A	0.9300
C2—C3	1.4054 (16)	C14—C15	1.3879 (16)
C3—C4	1.3865 (18)	C15—C16	1.3906 (16)
C3—H3A	0.9300	C15—H15A	0.9300
C4—C5	1.4027 (19)	C16—H16A	0.9300
C4—H4A	0.9300	O1W—H2W1	0.8477
C5—C6	1.3895 (19)	O1W—H1W1	0.8182
			
C7—O1—C8	105.33 (8)	C1—C8—C9	128.15 (10)
C9—N1—N2	116.97 (9)	O1—C8—C9	119.35 (9)
C9—N1—H1N1	122.2 (12)	O2—C9—N1	124.56 (10)
N2—N1—H1N1	120.5 (12)	O2—C9—C8	119.00 (10)
C10—N2—N1	116.14 (9)	N1—C9—C8	116.41 (9)
C8—C1—C2	105.98 (10)	N2—C10—C11	119.96 (10)
C8—C1—H1A	127.0	N2—C10—H10A	120.0
C2—C1—H1A	127.0	C11—C10—H10A	120.0
C7—C2—C3	118.90 (10)	C12—C11—C16	119.23 (12)
C7—C2—C1	105.87 (9)	C12—C11—C10	119.59 (12)
C3—C2—C1	135.23 (11)	C16—C11—C10	121.17 (11)
C4—C3—C2	118.16 (11)	C13—C12—C11	121.05 (12)
C4—C3—H3A	120.9	C13—C12—H12A	119.5
C2—C3—H3A	120.9	C11—C12—H12A	119.5
C3—C4—C5	120.93 (11)	C14—C13—C12	118.66 (11)
C3—C4—H4A	119.5	C14—C13—H13A	120.7
C5—C4—H4A	119.5	C12—C13—H13A	120.7
C6—C5—C4	122.31 (12)	C13—C14—C15	121.45 (11)
C6—C5—H5A	118.8	C13—C14—Br1	120.14 (9)
C4—C5—H5A	118.8	C15—C14—Br1	118.41 (9)
C7—C6—C5	115.40 (12)	C14—C15—C16	119.56 (11)
C7—C6—H6A	122.3	C14—C15—H15A	120.2
C5—C6—H6A	122.3	C16—C15—H15A	120.2
O1—C7—C6	125.38 (10)	C15—C16—C11	119.98 (11)
O1—C7—C2	110.34 (9)	C15—C16—H16A	120.0
C6—C7—C2	124.27 (10)	C11—C16—H16A	120.0
C1—C8—O1	112.45 (9)	H2W1—O1W—H1W1	116.8
			
C9—N1—N2—C10	175.83 (10)	N2—N1—C9—O2	0.50 (15)
C8—C1—C2—C7	1.26 (13)	N2—N1—C9—C8	178.34 (9)
C8—C1—C2—C3	−178.77 (14)	C1—C8—C9—O2	9.58 (17)
C7—C2—C3—C4	0.58 (18)	O1—C8—C9—O2	−173.05 (9)
C1—C2—C3—C4	−179.38 (13)	C1—C8—C9—N1	−168.39 (11)
C2—C3—C4—C5	0.73 (19)	O1—C8—C9—N1	8.97 (14)
C3—C4—C5—C6	−0.7 (2)	N1—N2—C10—C11	−178.76 (9)
C4—C5—C6—C7	−0.66 (19)	N2—C10—C11—C12	176.26 (11)
C8—O1—C7—C6	−178.42 (11)	N2—C10—C11—C16	−3.47 (17)
C8—O1—C7—C2	0.63 (11)	C16—C11—C12—C13	2.60 (18)
C5—C6—C7—O1	−178.99 (10)	C10—C11—C12—C13	−177.13 (11)
C5—C6—C7—C2	2.09 (18)	C11—C12—C13—C14	−1.58 (17)
C3—C2—C7—O1	178.84 (10)	C12—C13—C14—C15	−0.80 (17)
C1—C2—C7—O1	−1.19 (12)	C12—C13—C14—Br1	178.62 (9)
C3—C2—C7—C6	−2.09 (18)	C13—C14—C15—C16	2.11 (17)
C1—C2—C7—C6	177.88 (11)	Br1—C14—C15—C16	−177.32 (9)
C2—C1—C8—O1	−0.94 (13)	C14—C15—C16—C11	−1.05 (17)
C2—C1—C8—C9	176.58 (10)	C12—C11—C16—C15	−1.26 (18)
C7—O1—C8—C1	0.21 (12)	C10—C11—C16—C15	178.47 (11)
C7—O1—C8—C9	−177.55 (9)		

### Hydrogen-bond geometry (Å, º)

**Table:**

*D*—H···*A*	*D*—H	H···*A*	*D*···*A*	*D*—H···*A*
O1*W*—H2*W*1···O2^i^	0.85	2.00	2.7932 (11)	157
O1*W*—H1*W*1···O2^ii^	0.82	2.10	2.8987 (11)	167
N1—H1*N*1···O1*W*^iii^	0.890 (17)	1.947 (18)	2.8108 (13)	163.4 (17)
C6—H6*A*···O2^iv^	0.93	2.53	3.3418 (18)	146

Symmetry codes: (i) *x*, −*y*+1, *z*−1/2; (ii) *x*, −*y*, *z*−1/2; (iii) *x*, *y*−1, *z*; (iv) *x*, −*y*−1, *z*−1/2.
